# Magnetic Force-driven in Situ Selective Intracellular Delivery

**DOI:** 10.1038/s41598-018-32605-w

**Published:** 2018-09-21

**Authors:** Ran Wang, Yu Ting Chow, Shuxun Chen, Dongce Ma, Tao Luo, Youhua Tan, Dong Sun

**Affiliations:** 1 Department of Biomedical Engineering, City University of Hong Kong, Hong Kong, China; 2 The Hong Kong Polytechnic University Shenzhen Research Institute, Shenzhen, China; 3 Department of Biomedical Engineering, The Hong Kong Polytechnic University, Hong Kong, China; 4 Shenzhen Research Institute of City University of Hong Kong, Shenzhen, 518057 China

## Abstract

Intracellular delivery of functional materials holds great promise in biologic research and therapeutic applications but poses challenges to existing techniques, including the reliance on exogenous vectors and lack of selectivity. To address these problems, we propose a vector-free approach that utilizes millimeter-sized iron rods or spheres driven by magnetic forces to selectively deform targeted cells, which in turn generates transient disruption in cell membranes and enables the delivery of foreign materials into cytosols. A range of functional materials with the size from a few nanometers to hundreds of nanometers have been successfully delivered into various types of mammalian cells *in situ* with high efficiency and viability and minimal undesired effects. Mechanistically, material delivery is mediated by force-induced transient membrane disruption and restoration, which depend on actin cytoskeleton and calcium signaling. When used for siRNA delivery, CXCR4 is effectively silenced and cell migration and proliferation are significantly inhibited. Remarkably, cell patterns with various complexities are generated, demonstrating the unique ability of our approach in selectively delivering materials into targeted cells *in situ*. In summary, we have developed a magnetic force-driven intracellular delivery method with *in situ* selectivity, which may have tremendous applications in biology and medicine.

## Introduction

Delivery of macromolecules of interest across cell membranes, such as nucleic acids, proteins, siRNAs, and membrane-impermeable drug compounds, into mammalian cells has extensive applications in both biological research and therapeutics^1,2^. Carrier-based and membrane disruption-based methods have been developed to overcome cell membrane barriers when introducing exogenous materials into cells^3^. The former methods package materials into carriers, including viruses and non-viral vectors, such as liposomes, peptides, and nanoparticles, and deliver them into living cells mainly through endocytosis. These methods have the potential to achieve intracellular delivery with high efficiency and throughput but no selectivity. The use of virus raises risks in chromosomal integration and limits it to delivery of nucleic acids^4,5^; nanoparticle-based delivery is limited by nonspecificity^6^. Carrier-based methods meet challenges in transfecting blood, immune, and primary cells. The limited combination of feasible carrier materials and cell types hampers their further applications.

Compared to carrier-based delivery, membrane disruption-based approaches hold the ability to deliver diverse materials into a broad range of cell types^3^. Living cells can be deformed to generate transient disruption in cell membranes, which allows the surrounding macromolecules to passively diffuse into cytoplasm^7^. This idea has been recently emerging as a promising alternative for intracellular delivery. However, their inherent limitations are the potential membrane damage and poor throughput. For example, membrane disruption induced by a single nanoneedle has been used for delivery of plasmid DNA but with low throughput^8^. With the advancement of nanotechnology and microfluidics, penetration of cell membranes through an array of nanowires^9^ or nanoneedles^10^ achieves delivery of various biomolecules with high throughput. Membrane deformation induced by narrow microfluidic channels has been used to deliver diverse materials^11–15^. Ultrasound cavitation permeabilizes cell membranes for intracellular delivery of molecules^16^. Electroporation has been adopted to deliver various biomolecules^17^. However, these techniques lack the ability to selectively deliver materials into targeted cells *in situ*. Microinjection^18,19^ and optical transfection^20^ can achieve highly selective delivery but at the cost of low throughput.

To address these problems, we proposed a magnetic force-driven approach in this study to achieve vector-free and selective *in situ* intracellular delivery. A wide range of materials were delivered into various types of mammalian cells and the delivery efficiency and cell viability were examined. The mechanisms of how materials pass through cell membranes and the influence of cytoskeleton and calcium on intracellular delivery were explored. The effects of delivered siRNAs on cellular functions were examined. Finally, the ability of our method to selectively deliver materials into targeted cells was demonstrated.

## Results

### Magnetic forces drive intracellular delivery with high efficiency and viability

**Figure 1:**
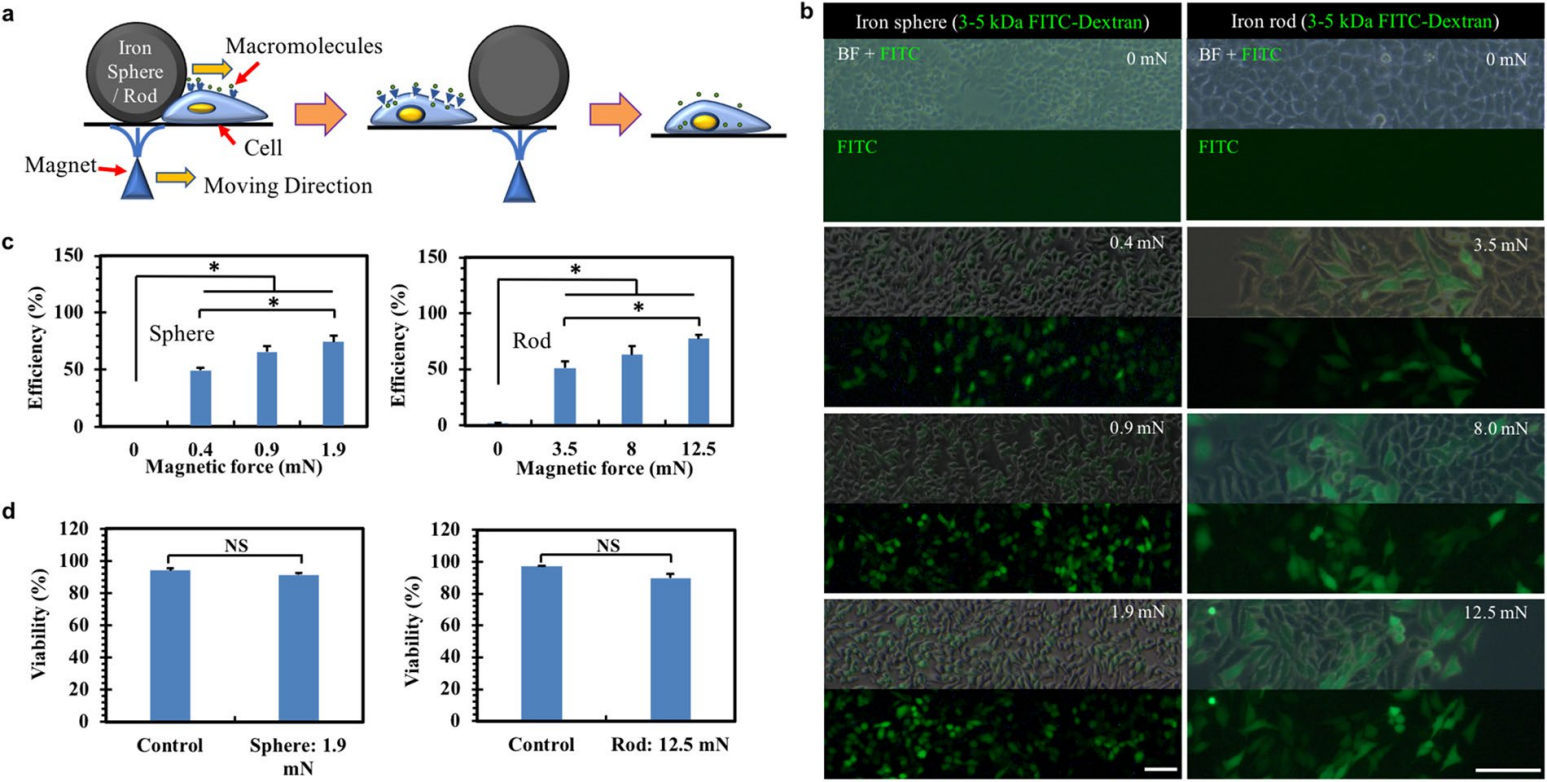
Magnetic force-driven intracellular delivery. **(a)** Schematic of the magnetic force-driven intracellular delivery method. An iron sphere/rod was driven by magnetic forces to deform living cells, which generated membrane disruption and facilitated the diffusion of exogenous materials into cytosols. For sake of convenience, the schematic is not drawn to scale. **(b)** The delivery of FITC-dextran into cells using iron sphere (left) or rod (right) depends on force magnitude. Scale bar: 100 µm. Various magnetic forces were used to actuate the sphere or rod, which deformed living cells in the presence of 3–5 kDa FITC-dextran, which was removed after 5 min. The fluorescence images were taken at 24 h after force application. BF: bright field. Quantification of the delivery efficiency **(c)** and cell viability **(d)** for both sphere (left) and rod (right). For sphere, the FITC-positive and PI-negative cells were counted from the fluorescence images. The efficiency and viability were calculated as the ratio of FITC-positive cells and PI-negative cells over the total cell number, respectively. For rod, flow cytometry assay was used to quantify the percentage of FITC-positive cells and PI-negative cells. Mean ± s.e.m (n = 3). *p < 0.05. NS: no significant difference.

In this study, only one iron sphere or rod was actuated by a ramped magnetic field generated by a customized micromanipulator-controlled magnet with a sharp pole tip (Fig. 1a and Supplementary Fig. S1). The actuated sphere/rod exerted forces onto the underlying cells for material delivery that could be modulated by adjusting the distance between the sphere/rod and the magnet (Fig. 1a and Suplementary Fig. S2). The motions were synchronized so that the trajectory of the sphere/rod could be controlled by the magnet. For sphere, a portion of cells underneath experience the force, thereby making it suitable for selective delivery, including cell pattern formation. For rod, a large number of cells experience the force, which can achieve efficient delivery.

To validate the approach, fluorescein isothiocyanate-labeled dextran (FITC-dextran; 3–5 kDa) was chosen to characterize the delivery efficiency, since it is membrane impermeable with the size at the nanometer scale similar to many proteins and siRNAs. To explore the effects of the size of sphere/rod, spheres with various diameters were tested. The data show that the sphere with 1 mm diameter achieved slightly high delivery efficiency, while sphere size had no effects on cell viability (Supplementary Fig. S3a and S3b). When the distance between the sphere and magnet was fixed, the magnetic force increased along with sphere size (Supplementary Fig. S3c). Then, the influence of driving speed of the sphere/rod was examined. The results show that the adopted speeds have no significant influence on delivery efficiency (all around 70%) and cell viability (Supplementary Fig. S4). The possible reason could be the fact that the highest driving speed or the shortest duration of force loading (a few ms) and the force we adopted might be enough to induce membrane disruption that mediated material delivery. Therefore, the sphere with 1 mm diameter at 6 mm/s was adopted in the rest of the study unless otherwise specified.

FITC-dextran was successfully delivered into human hepatocellular carcinoma (HCC) cells HepG2 on the trajectory of the actuated sphere/rod (Fig. 1b). When the magnetic force was increased from 0 mN to 1.9 mN for the sphere and from 0 mN to 12.5 mN for the rod, the delivery efficiency was increased from 0% to 74% and from 2% to 77%, respectively (Fig. 1c), showing that the intracellular delivery depends on the magnitude of magnetic force. It may be due to the fact that larger forces cause higher levels of membrane disruption that could enhance material delivery. In addition, the delivery efficiency is related to molecule concentration under a given force (Supplementary Fig. S5), which may be due to the fact that molecules at higher concentrations have better chances to pass across the disrupted membranes. The contact area between the sphere/rod and cells could be estimated as the area of cells expressing the fluorescence of delivered materials (~0.05 mm^2^ for the sphere with 1 mm diameter). Therefore, the contact stress was calculated to be 40 kPa for sphere (1.9 mN), which was at the similar level of 15 kPa solid stress in tissues^21^ but much higher than hemodynamic shear stress in vasculature (within 3 Pa)^22^. Note that the duration of force application in this approach was short (a few milliseconds).

We further examined whether the forces exerted on cells in our approach influenced cell functions, including viability, proliferation, and stem cell properties. For both sphere and rod, high magnetic forces (1.9 mN for sphere and 12.5 mN for rod) led to ~90% of cell viability (Fig. 1d and Supplementary Fig. S6a) and had no significant effects on the proliferation of primary human mesenchymal stem cells (MSCs) (Supplementary Fig. S6b). Physical forces have the potential to elicit stem cell differentiation^23–25^. To explore this effect, the magnetic force at 12.5 mN for the rod was exerted on MSCs. The data show that the applied forces had no detectable effects on the expression of stem cell marker CD44^26^ (Supplementary Fig. S6c), suggesting that the forces adopted in our approach have minimal effects on stemness. In addition, cell membranes appear to be fully recovered at 3 h after force loading (Supplementary Fig. S6d), suggesting that the exerted force during the intracellular delivery does not induce permanent defects in membrane permeability. Therefore, these data suggest that our proposed method could efficiently deliver macromolecules into targeted cells with minimal undesired effects on cell functions.

### Intracellular delivery is mediated by cytoskeleton- and calcium signaling-dependent membrane disruption and recovery

**Figure 2:**
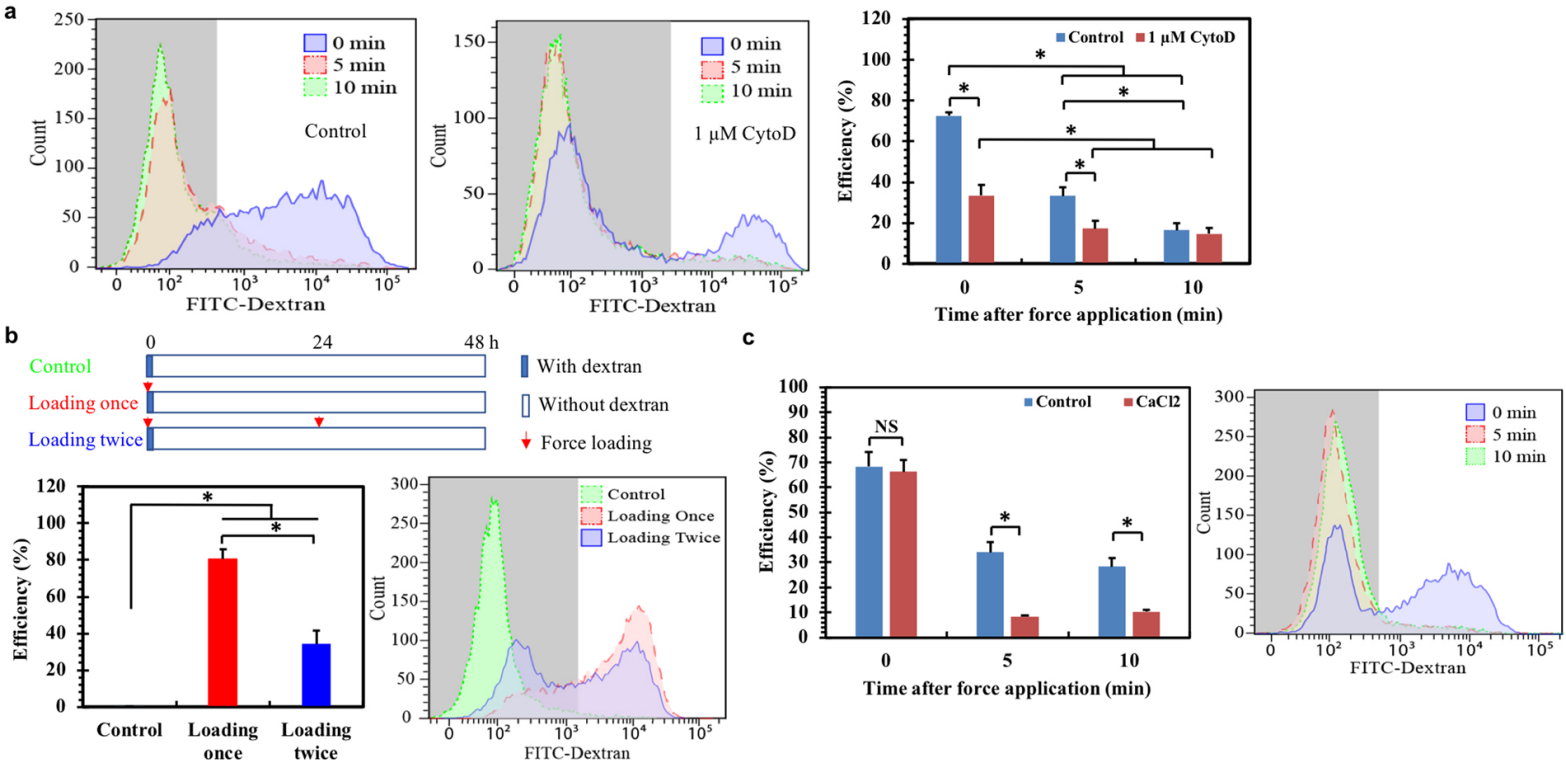
Intracellular delivery is mediated by cytoskeleton- and calcium signaling-dependent membrane disruption and recovery. **(a)** Intracellular delivery is mediated by cytoskeleton-dependent transient membrane disruption. Left and middle: representative flow cytometry figures of HepG2 cells expressing FITC fluorescence when cells were pre-treated with 1 µM cytochalasin D (CytoD) or DMSO (Control) for 3 h before loading by the rod at 12.5 mN. 3–5 kDa FITC-dextran (1 mg/ml) was added at 0, 5, and 10 min post force application. Right: quantification of the delivery efficiency by flow cytometry analysis (n = 3). **(b)** The delivery of exogenous materials across disrupted cell membranes is reversible under repeated force application. Top: the experimental protocol; bottom left: quantification of the delivery efficiency; bottom right: representative flow cytometry figure. “Control”: HepG2 cells were cultured in the presence of 3–5 kDa FITC-dextran for 5 min without force application. “Loading once”: cells were loaded once by the rod in the presence of FITC-dextran for 5 min at 0 h. “Loading twice”: cells were loaded twice by the rod, in which the first time was in the presence of FITC-dextran for 5 min at 0 h and the second time was in the absence of FITC-dextran at 24 h. All samples were retrieved at 48 h for flow cytometry analysis (n = 3). All the loading forces were 12.5 mN and the dextran concentration was 1 mg/ml. **(c)** Calcium signaling facilitates the recovery of force-induced membrane disruption. Left: quantification of the delivery efficiency (n = 3); right: representative flow cytometry figure. HepG2 cells were deformed by the rod in the presence or absence (Control) of 0.2 µM CaCl2 with the addition of 2000 kDa FITC-dextran at 0, 5, and 10 min post force application, respectively. The delivery efficiency was measured after 24 h by flow cytometry analysis. Mean ± s.e.m.; *p < 0.05.

The influence of force magnitude on delivery efficiency suggests that material delivery may be achieved via force-induced transient membrane disruption or re-organization that enables passive diffusion of the surrounding macromolecules into cells. To test this idea, the intracellular distribution of the delivered materials was examined. The results show that tetramethylrhodamine (TRITC)-dextran internalized through endocytosis was mainly localized in lysosomes, while the molecules delivered via our method were evenly distributed within cytoplasm but not lysosome (Supplementary Fig. S7). These findings suggest that magnetic force driven intracellular delivery may be not mediated through endocytosis. To explore the underlying mechanisms, FITC-dextran was added into the medium at 0, 5, and 10 min after cells were loaded, respectively. The data show that regardless of dextran concentration (Supplementary Fig. S8), the delivery efficiency is 72% at 0 min but dramatically decreases to 33% at 5 min and to 16% at 10 min (Fig. 2a). Note that the size of 3–2000 kDa dextran is less than 60 nm and it could not freely diffuse into cells within 5 min without force application^27^ (Fig. 2b). These findings suggest that cell membranes may be disrupted rapidly under forces and progressively recovered within 10 min. Continuous decrease in delivery efficiency may be due to the increased restoration level of disrupted cell membranes.

To further demonstrate that membrane disruption mediates intracellular delivery, cells were loaded by the iron rod in the presence of FITC-dextran for the first time (Fig. 2b). Dextran was removed after 5 min and replaced by fresh medium. After 24 h, these cells were subject to the second loading without dextran. Flow cytometry analysis was conducted at 48 h after the first loading. The data show that the percentage of FITC-positive cells reaches 80% when loading once but significantly decreases to ~34% after the second loading without dextran (Fig. 2b). This is probably due to the fact that molecules diffuse along the concentration gradient. The first loading may transiently create disruption in cell membranes that facilitates the influx of dextran into cytosols, since the molecule concentration is high in the medium. After removal, the dextran concentration becomes low in medium but relatively high in cytosols. The membrane disruption induced by the second loading mediates the efflux of dextran from cells so that the delivery efficiency is decreased (Fig. 2b). Note that repetitive force loading has no effects on cell viability (Supplementary Fig. S9). These findings suggest that rapid membrane disruption and recovery probably underpin magnetic force-driven intracellular delivery.

To explore the roles of cytoskeleton, HepG2 cells were pre-treated with actin inhibitor cytochalasin D and then loaded by the rod with the addition of FITC-dextran at 0, 5, and 10 min after loading, respectively. The data show that actin inhibition dramatically decreases the delivery efficiency from 72% to 33% and from 33% to 17% at 0 and 5 min, respectively (Fig. 2a). Actin disassembly blocks the further decrease in delivery efficiency at 10 min, suggesting that actin disruption may retard membrane recovery. The possible explanation could be that as the structural basis, cytoskeleton influences the transmission of forces inside cells and thus the generation of membrane disruption and recovery. These findings suggest that actin cytoskeleton may be critical in force-induced membrane disruption and restoration, which in turn affect intracellular delivery. It is known that calcium signaling is important in membrane repair^28^. To examine its roles in intracellular delivery, FITC-dextran was added in the presence of 0.2 µM CaCl2 at 0, 5, and 10 min after loading, respectively. The data show that different calcium concentrations barely change the delivery efficiency at 0 min (Supplementary Fig. S10a). Calcium signaling significantly decreases the delivery efficiency at 5 and 10 min (Fig. 2c and Supplementary Fig. S10b), suggesting that force-induced membrane disruption may be gradually restored in a calcium-dependent manner. A plausible explanation is that calcium may facilitate membrane restoration so that more transient disruptions generated in cell membranes may be recovered at 5 and 10 min in the presence of calcium, which in turn decreases material delivery and the efficiency.

### Various materials are delivered into mammalian cells across cell types

**Figure 3:**
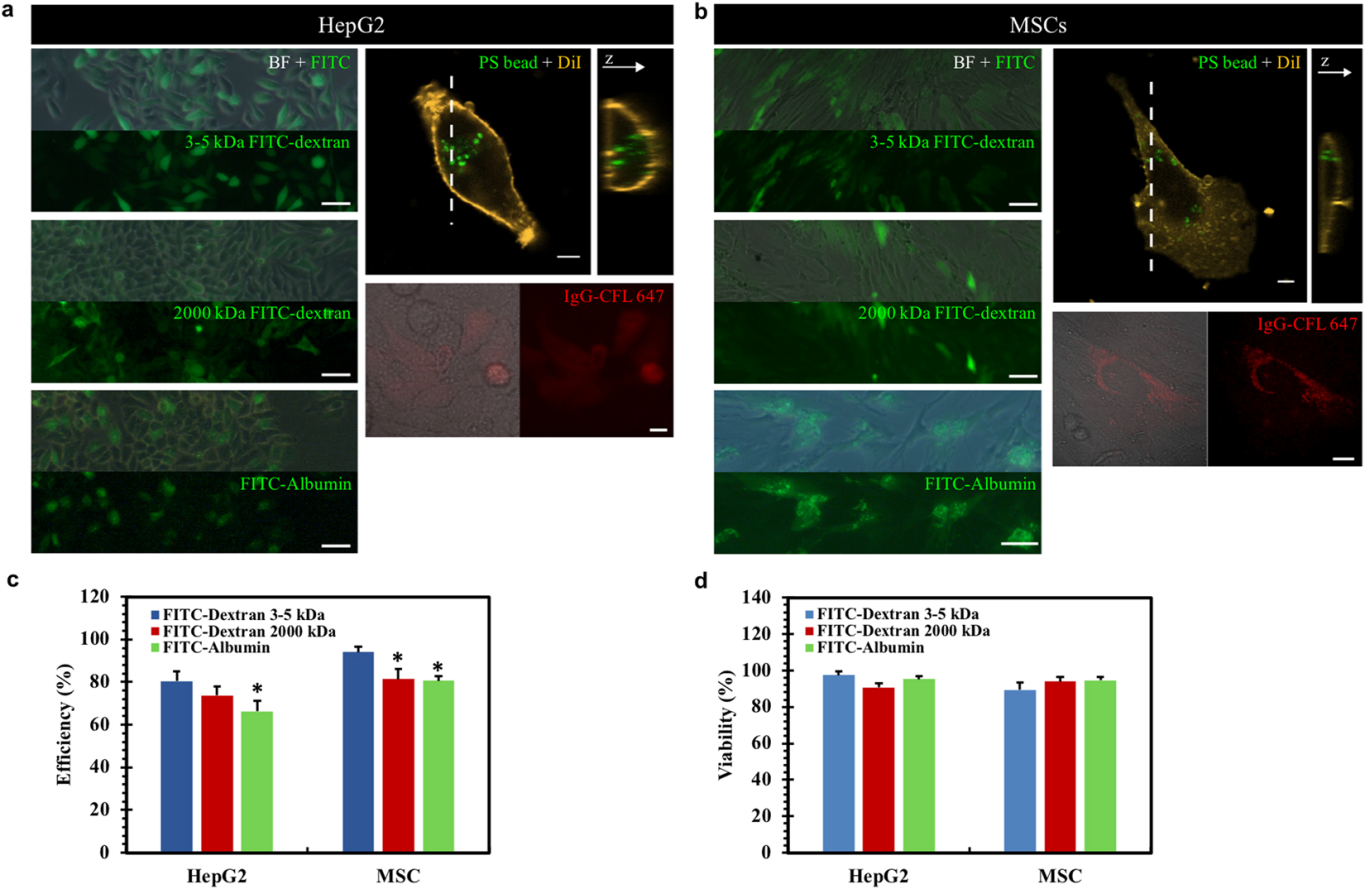
Various materials are delivered into mammalian cells across cell types. A wide range of molecules with the size from a few nanometers to hundreds of nanometers can be delivered into HepG2 cells **(a)** and primary MSCs **(b)** with high efficiency and viability. Cells were deformed with the rod in the presence of 3–5 or 2000 kDa FITC-dextran, FITC-albumin (scale bars: 100 µm), polystyrene (PS) beads (200 nm in diameter; scale bar: 10 μm), and antibody IgG-CFL 647 (scale bar: 20 μm), respectively. Fluorescence images were taken after 24 h. Quantification of the delivery efficiency **(c)** and cell viability **(d)**. All the cells in (a) and (b) were stained with PI and then used to quantify the efficiency and viability. For cells with PS beads, the Y-Z cross-section images were reconstructed from Z-series fluorescent images of the cell. The sectioning plane was indicated by the dashed line. Cell membrane was counterstained with DiI. *p < 0.05, representing the significant difference between “FITC-Dextran 3–5 kDa” and other groups.

We then asked whether our proposed approach could be used as a general method for intracellular delivery. To this end, material delivery was conducted in various mammalian cells (Fig. 3 and Supplementary Fig. S11), including HCC cells HepG2 and MHCC97-L, human dermal fibroblasts, neonatal (HDFn), human cervical cancer cells HeLa, preosteoblast MC3T3 E1, and primary human MSCs. The data show that macromolecules were successfully delivered into all these cells with high efficiency and viability (Supplementary Fig. S11), suggesting our magnetic force-driven approach is applicable across cell types. We further examined whether our method could deliver various types of materials into cells. The data show that FITC-dextran with both small (3–5 kDa) and large (2000 kDa) molecular weight could be successfully delivered into HepG2 cells and primary MSCs that are usually difficult to be transfected (Fig. 3a,b; top left). The delivery efficiency is lower for large molecules (80% versus 74% in HepG2; 94% versus 81% in MSCs) (Fig. 3c), probably due to the increasing molecule size, which confirms that the size-dependent delivery may be mediated by transient membrane disruption. Fluorescence-labeled bovine albumin (FITC-albumin, ~66 kDa) was also delivered with the efficiency of 66% and 80% for HepG2 and MSCs, respectively (Fig. 3a,b; bottom left; Fig. 3c). Further, large polystyrene beads (200 nm in diameter) could also be delivered (Fig. 3a,b; top right), where 3D reconstruction of the Z-series scanning shows that the delivered beads are inside the cells. In addition, the fluorescence-labeled secondary antibody (donkey anti-goat IgG-CFL 647) was successfully delivered (Fig. 3a,b; bottom right), demonstrating that this approach may be used for live cell immunostaining. Moreover, efficient delivery of various materials into mammalian cells does not compromise cell viability (Fig. 3d). All these findings demonstrate that our magnetic force-driven method is able to deliver various macromolecules into different types of mammalian cells.

### Delivered siRNAs inhibit cancer cell proliferation and migration

**Figure 4:**
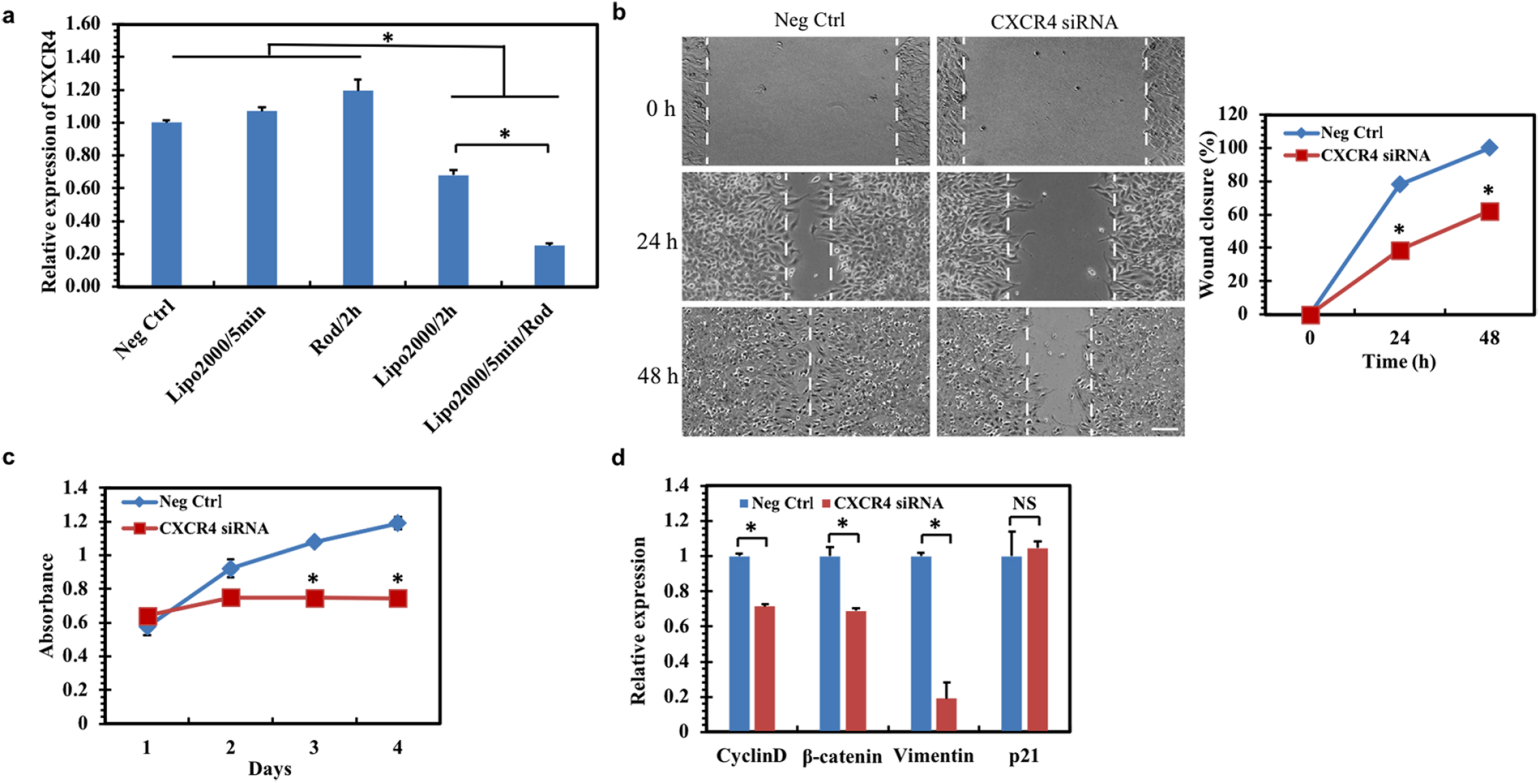
Delivered CXCR4 siRNA inhibits gene expression and cancer cell proliferation and migration. **(a)** The delivered CXCR4 siRNA effectively silences gene expression. HepG2 cells were incubated with the mixture of lipofectamine 2000 and negative control siRNA (Neg Ctr) for 2 h, or of lipofectamine 2000 and CXCR4 siRNA for 2 h (Lipo2000/2 h) or 5 min (Lipo2000/5 min) without force application, or of CXCR4 siRNA for 2 h with force application by the rod but no lipofectamine 2000 (Rod/2 h), or of lipofectamine 2000 and CXCR4 siRNA for 5 min with force application by the rod (Lipo2000/5 min/Rod). The expression of CXCR4 was quantified by quantitative RT-PCR after 48 h. **(b)** Delivered CXCR4 siRNA inhibits cancer cell migration. Left: representative wound healing figures; right: quantification of wound closure at the indicated times. **(c)** Delivered CXCR4 siRNA inhibits cancer cell proliferation. The CXCR4 knockdown cells in (b) were cultured in petri dishes and used for proliferation analysis by MTT assay at day 1, 2, 3, and 4, respectively. **(d)** Silencing CXCR4 inhibits the expressions of β-catenin, vimentin, cyclin D but not p21.

We further examined whether the delivered materials could be functional in regulating cellular functions. To this end, siRNAs were delivered as an example to target CXCR4, the receptor of cytokine CXCL12, which is overexpressed in various cancer types and crucial in cancer cell proliferation, migration, and metastasis^29^. The data show that the liposome-based transfection inhibits CXCR4 expression by 32% when the liposome-siRNA mixture was incubated with cells for 2 h (Fig. 4a). If the incubation time was 5 min or cells were loaded without liposome, no knockdown effects were detected. In contrast, our intracellular delivery approach rapidly delivered siRNA mixture into cells within 5 min, which leads to the knockdown of CXCR4 expression by 75% (Fig. 4a), much more efficient than the liposome-based transfection. We then tested whether the delivered siRNAs influenced cellular functions, including migration and proliferation. The wound healing assay shows that the delivered CXCR4 siRNA significantly suppresses the wound closure (Fig. 4b), suggesting that silencing CXCR4 effectively inhibits cell migration, which is consistent with the findings that CXCL12/CXCR4 signaling enhances the migration and invasion of cancer cells^30^. The inhibitory effects on cell migration may be mediated by suppressing the downstreams of CXCR4, including β-catenin and vimentin^31^ (Fig. 4d). The MTT data show that silencing CXCR4 significantly inhibits cell proliferation (Fig. 4c), which is probably due to the downregulation of its downstream effectors cyclin D and β-catenin^31^ (Fig. 4d). Note that silencing CXCR4 has no significant effects on p21 expression (Fig. 4d). These data suggest that the materials are functional after intracellular delivery.

### Cell patterns are generated by selectively delivering materials into targeted cells *in situ*

**Figure 5:**
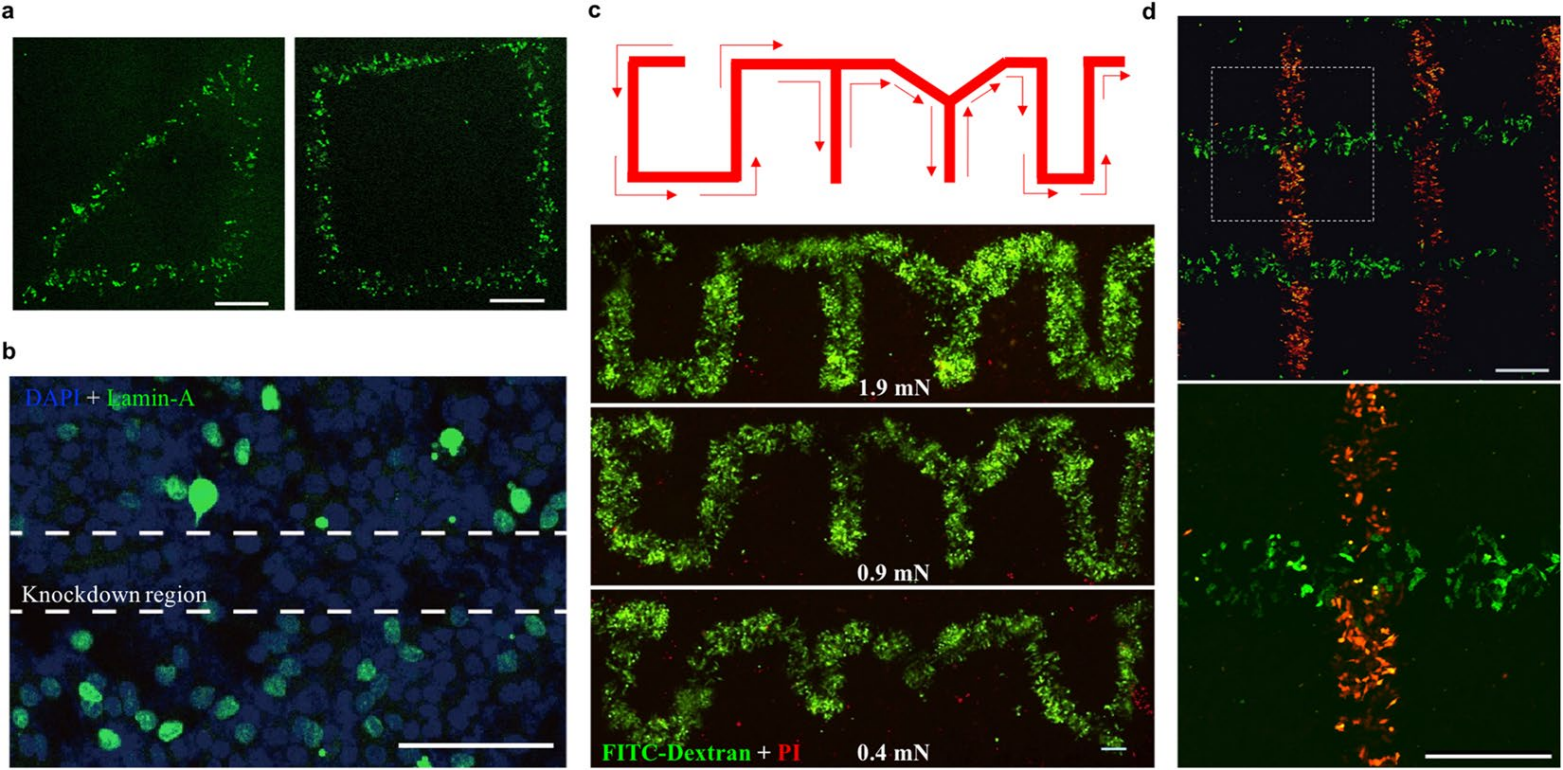
Cell patterns are generated by selectively delivering materials into targeted cells *in situ*. **(a)** Formation of cell patterns with various shapes. The sphere was actuated by the magnetic force at 1.9 mN under robotic control to follow the trajectory of triangle or square. HepG2 cells were loaded in the presence of 3–5 kDa FITC-dextran for 5 min. The fluorescence images were captured after 24 h. Scale bar: 500 µm. **(b)** Cell pattern formation after siRNA delivery. Lamin A siRNA was first incubated with lipofectamin 2000 for 10 min and the mixture was then delivered by the rod into MHCC-97L cells within 5 min. The medium was then replaced by fresh cell culture medium and cells were cultured for 72 h before immunostaining was conducted for the analysis of lamin A expression (green). Cell nuclei were counterstained with DAPI (blue). The region between the dashed lines was on the trajectory of the actuated sphere, showing the efficient knockdown of lamin A. Scale bar: 100 µm. **(c)** Formation of “CITYU” cell pattern. Top: schematic of the sphere trajectory; bottom: fluorescence images of the generated cell patterns. HepG2 cells were loaded under magnetic forces at 0.4, 0.9, and 1.9 mN by the sphere in the presence of 3–5 kDa FITC-dextran, respectively. The fluorescence images were taken after 24 h. Scale bar: 200 µm. **(d)** Formation of two-color cell patterns. Top: large field of view; bottom: enlarged view of the indicated area in the top one. HepG2 cells were loaded by the sphere in the horizontal direction with 2000 kDa FITC-dextran. After 10 min, the cells were loaded again in the direction perpendicular to the previous trajectory in the presence of fresh culture medium with 4.4 kDa TRITC-dextran. The fluorescence images were captured after 24 h. Scale bar: 500 µm.

For sphere, only a portion of cells on the trajectory are subject to forces while other cells remain intact. We then explored whether selective intracellular delivery especially the formation of cell patterns could be achieved. To this end, the motion of the sphere was precisely controlled by the magnet tip mounted on the micromanipulator that could be robustly controlled through the motorized stage (Supplementary Fig. S1). Thus, the sphere could closely follow a pre-designed trajectory, thereby loading cells in customized locations and enabling intracellular delivery of materials into targeted cells that form specific cell patterns. To demonstrate this ability, the sphere was actuated to follow various trajectories (e.g., triangle and square) and deform cells in the presence of FITC-dextran (Fig. 5a). Macromolecules were efficiently delivered to generate various cell patterns with differential complexities. Lamin-A siRNAs were also utilized for intracellular delivery. The expression of lamin-A protein on nuclear membranes is significantly decreased only in the cells on the trajectory of the sphere (Fig. 5b), making it suitable to generate cell patterns.

The sphere was then controlled to follow a pattern of “CITYU”. FITC-dextran was delivered selectively to the targeted cells that create the designed patterns (Fig. 5c). Since the material delivery depends on force magnitude (Fig. 1c), the pattern quality is related to the magnetic forces exerted on the sphere. The data show that the generated patterns well represented the designed ones at 1.9 mN force but were distorted at 0.4 mN force (Fig. 5c). This might be due to the facts that the delivery efficiency is poor and the sphere is unable to strictly follow the magnet trajectory under low forces. Finally, HepG2 cells were loaded by the sphere in the horizontal direction with FITC-dextran, and loaded again after 10 min in another trajectory perpendicular to the previous one in the fresh medium with TRITC-dextran (Fig. 5d). Both macromolecules were delivered efficiently and selectively to cells at different locations, which generated the two-color patterns (Fig. 5d). All these findings demonstrate that our magnetic force-driven intracellular delivery approach holds the unique ability to selectively deliver different materials into targeted cells *in situ* and generate desired cell patterns.

## Discussion

In this study, we propose a magnetic force-driven and vector-free method for *in situ* selective intracellular delivery, which utilizes an actuated iron sphere or rod to deform living cells and deliver the surrounding macromolecules into targeted cells. A wide range of materials from a few nanometers to hundreds of nanometers, including macromolecules, proteins, siRNAs, antibodies, and nanoparticles, have been successfully delivered into various types of mammalian cells with high efficiency while maintaining high viability. Living cells have the ability to sense and respond to mechanical forces^23–25,32^. However, the side effects of exerted forces on cell viability, proliferation, and stemness properties are minimal in our proposed approach, likely due to the very short force application (a few milliseconds). The size of sphere/rod affects magnetic force and delivery efficiency but not cell viability. The adopted driving speed of sphere/rod has no obvious effects on the delivery efficiency and cell viability. The application of multiple spheres/rods in intracellular delivery may be explored in the future. Compared to traditional transfection methods, this approach can efficiently and rapidly deliver various types of materials into those hard-to-transfected cells, such as primary MSCs, and greatly enhance the transfection efficiency. Further, when used for siRNA delivery, the delivered CXCR4 siRNAs silence gene expression, much more efficient than the traditional liposome-based method. Moreover, CXCR4 siRNAs effectively suppress cell migration and proliferation by inhibiting its downstream genes and signaling, suggesting that the materials are functional inside cells after delivery.

The delivery efficiency depends on the magnitude of exerted forces and the force-induced transient disruption in cell membranes enables the delivery of exogenous materials into cytosols. Cell membranes are disrupted rapidly (probably in seconds to minutes) and partially recovered in several minutes (less than 5 min). Membrane disruption and recovery depend on intact cytoskeleton and calcium signaling. Force-mediated material delivery is completed within 5 min, which is not long enough for endocytosis, as the time required for this process is usually more than half an hour^33,34^. This is consistent with our finding that there is negligible intracellular delivery when co-culturing cells with materials for 5 min without force loading. The cross-membrane material delivery is reversible, supported by the findings that the first loading induces the influx of foreign materials into cytosols and the second loading leads to the efflux of those delivered materials out of cytosols. Further, the materials delivered through our method are mainly distributed within cytoplasm, while those internalized via endocytosis are localized in lysosome, suggesting magnetic force driven intracellular delivery may be not medicated through endocytosis. Collectively, the delivery mechanism in this study appears to be passive diffusion of materials across disrupted membranes rather than active transport, including endocytosis.

The existing techniques for intracellular delivery have the ability to deliver materials into a large population of cells at high throughput but lack of selectivity^1–4,6,9,10,12,16,17^. Several approaches have been developed to achieve selective delivery of materials into spatially specified cells but with poor throughput^18–20^. In comparison, our proposed method holds the unique ability to selectively deliver exogenous materials into spatially targeted living cells *in situ* with relatively high throughput at the same time. The intracellular delivery in this study is mediated through force-induced membrane disruption, which is similar to other membrane disruption-based approaches induced by various techniques, including needle puncture, microfluidic squeezing, electroporation, and ultrasound^7,12,16,17^. Note that the interactions between sphere/rod and cells involve both vertical and lateral forces, which may be important in inducing membrane disruption and intracellular delivery. It is challenging to decouple the effects of both forces, which needs to be rigorously studied in the future.

With the assistance of advanced robotic technology^35^, our proposed approach provides an alternative method to robustly deliver distinct functional materials into cells at different locations within an engineered tissue, which in turn empowers these cells to hold unique phenotypes or functions and generates various cell patterns. This technique will have potential in many biologic research and therapeutic applications, including tissue engineering^36^ and cell-based therapy^37^. One of the challenges is to instruct stem cells at different locations to differentiate into distinct lineages^38^. Our approach can provide a powerful tool to deliver different macromolecules as instructive cues to guide the differentiation of stem cells at distinct locations within the same tissue. Another potential application is to deliver specific macromolecules into damaged or abnormal cells within a tissue *in situ* for therapy, which is difficult to be achieved using the existing methods. In addition to the traits of vector-free and selective intracellular delivery *in situ* and the ability to deliver materials into hard-to-transfect cells, our proposed method is simple, economic, and ready to be integrated into conventional experimental systems for wide applications. Note that our intracellular delivery approach is currently limited to deliver materials into cells on planar substrates. It is challenging to directly extend it from two-dimensional into three-dimensional tissues for *in vivo* applications, which is worth future exploration.

## Method

### Cell culture

Cells were cultured with DMEM (HepG2, MHCC-97L, HDFn, HeLa, and MC3T3 E1) or alpha-MEM (primary MSCs) supplemented with 10% FBS and 1% penicillin–streptomycin in a humidified atmosphere of 5% CO_2_ at 37 °C.

### Magnetic force-driven intracellular delivery

The iron sphere was bearing ball and rinsed with ethanol and stored in oil to prevent oxidation. The iron rod was home-made by sealing a rectangular iron into a 1 mm diameter glass capillary (B100-58-10, Sutter Instrument) with PDMS. Before and after the experiments, the iron sphere and rod were sterilized with 75% ethanol and rinsed with PBS. Only one sphere or rod was used in this study. The experiments were conducted in a 3D robotic micromanipulation system, which consisted of motorized XYZ stages (Supplementary Fig. S1). After changing the culture medium to delivery buffer (PBS mixed with desired delivery materials), a sterilized iron sphere (0.6–1.5 mm in diameter) or rod (1 mm in diameter and 10 mm in length) was transferred into the petri dish with cells placed on the Z-axis table. A magnet with a sharp pole tip fixed on the XY stage generated the effective magnetic field to actuate the sphere/rod that was aligned with the magnet tip by moving the dish close to the magnet. The distance between the tip and the sphere/rod was precisely controlled to adjust magnetic forces according to the calibrated force-distance relationships (See supplementary Fig. S2). The sphere/rod was driven in the XY plane under computer control by magnetic forces, thereby exerting forces on the underlying cells. After experiments, cells were rinsed twice with PBS and cultured in regular culture medium. Delivered materials were listed as Supplementary Table S1.

### Quantification of delivery efficiency and cell viability

Due to the difference in throughput between sphere and rod, different methods were adopted to quantify the delivery efficiency and cell viability. The treated cells were incubated with propidium iodide (PI, 500 nM) for 5 min. For sphere, fluorescence images were captured for analysis by ImageJ. Only the cells on the sphere trajectory were analyzed, in which cells expressing the fluorescence the delivered material carries (e.g., FITC) and not expressing PI fluorescence (red) were considered as the successfully delivered cells and viable cells, respectively. The delivery efficiency was determined as the ratio of FITC-positive (or fluorescence that the delivery material carries) cell number to the total cell number. Cell viability was calculated as the ratio of PI-negative cell number to the total cell number. Cell numbers were estaimated by counting cell areas. For rod, all the cells in the dish were treated and collected after 24 h and the delivery efficiency and viability were measured by flow cytometry assay (BD FACSVerse). When the fluorescence intensity was higher than a threshold value, the cells were considered to be fluorescence positive or successfully delivered. This value was determined based on the intensity of the control group.

### Quantitative RT-PCR

Total mRNAs were extracted from cells with Trizol reagent (Invitrogen) for cDNA synthesis using iScriptTM cDNA synthesis kit (Cat No170–8890, Bio-Rad, USA) following the manufacturer’s protocol. Real-time quantitative PCR amplification was performed with SsoAdvanced SYBR Green supermix kit (Cat No. 1725260, Bio-Rad, USA) in CFX96 Real-time System (Bio-Rad, USA). The specific primers used in this study were listed in Supplementary Table S2. Cyclin D, p21, and vimentin primers were described in references^39–41^. Additional primer sequences were obtained from the Primer Bank^42^.

### Wound healing assay

1.5 × 10^6^ cells were cultured in 6-well plates overnight until confluent. A 200 µl pipette tip was used to make a straight scratch. The suspended cells were removed by gentle rinse and images of the scratch were acquired as baseline. The medium was then replaced and images of the same location were obtained every 24 h for the next 3 days.

### MTT assay

3000 cells were seeded per well in 96 well plates in normal cell growth media. MTT assay was performed using MTT Cell Proliferation and Cytotoxicity Assay Kit (Sangon Biotech, China) according to the manufacturer’s protocol. The absorbance at 570 nm was measured by SpectraMax M5e Microplate Reader (Molecular Devices, CA) to estimate MTT-formazan production after culture for 24, 72, and 96 h, respectively.

### Immunofluorescence staining

Cells were fixed in 4% paraformaldehyde for 15 min, permeabilised in 0.25% Triton X-100 for 10 min and then blocked with 4% bovine serum albumin for 1 h at room temperature. Cells were incubated with primary antibody diluted in blocking solution for 2 h at room temperature, rinsed with PBS, incubated with secondary antibodies for 1 h, and rinsed with PBS again. Cell nuclei were counterstained with DAPI. The primary antibodies included anti-CD44 (Beyotime, 1:100) for MSCs and anti-Lamin A (Abcam, 1:100) for MHCC97L cells.

### Statistical analysis

At least three independent experiments were conducted for all experiments. Two-tailed Student t-test was used for statistical analysis.

## Electronic supplementary material

Supplementary information
