# Supplementary material for: Magnetic Force-driven in Situ Selective Intracellular Delivery
Source: Sci Rep. 2018 Sep 21;8:14205. doi: 10.1038/s41598-018-32605-w (PMC6155070; doi:10.1038/s41598-018-32605-w)
Supplement: Supplementary file 1 — Supplementary information [file 41598_2018_32605_MOESM1_ESM.docx]

**Supplementary information**

Magnetic Force-driven in Situ Selective Intracellular Delivery

Ran Wang^1†^, Yu Ting Chow^1†^, Shuxun Chen^1†^, Dongce Ma^1^, Tao Luo^1^, Youhua Tan^2,3*^, and Dong Sun^1,4*^

^1^Department of Biomedical Engineering, City University of Hong Kong, Hong Kong, China.

^2^The Hong Kong Polytechnic University Shenzhen Research Institute, Shenzhen, China. ^3^Department of Biomedical Engineering, the Hong Kong Polytechnic University, Hong Kong, China.

^4^Shenzhen Research Institute of City University of Hong Kong, Shenzhen 518057, China.

^†^These authors contributed equally to this work.

*Correspondence should be addressed to:

Youhua Tan ([youhua.tan@polyu.edu.hk](mailto:youhua.tan@polyu.edu.hk)), Dong Sun ([medsun@cityu.edu.hk](mailto:medsun@cityu.edu.hk))

**List of Contents**

1. **Additional material and methods (page 2)**
2. **Supplementary Table 1 and 2, Supplementary Figure S1-S11 (page 3-16)**

**Additional material and methods**

**Magnetic force calibration.**

The magnet was placed on a mass scale, which was fixed on the XY stage to calibrate the magnetic force in the experimental system (Supplementary Fig. S2). When the iron sphere/rod was coupled with the magnet tip, the magnetic force between them partially balanced the gravitational force of the magnet, which was measured by the scale. Therefore, the magnetic force could be calculated as the decreased amount of the readout of the scale before and after the interaction. The sphere/rod-magnet distance was varied through the Z-axis table to calibrate the relationship between magnetic forces and the sphere/rod-magnet distances.

**Simulation of magnetic forces between iron sphere/rod and magnet.**

The iron sphere/rod-magnetic tip models were constructed in Comsol Multiphysics magnetic field without the current module. In the sphere-magnetic tip model, the iron sphere had a diameter of 1 mm and magnetic susceptibility of 4000, while the magnetic tip had a remnant magnetic field of 0.335T in Z-direction. The magnetic tip was assumed to be a cone with 1.3 mm in height and 0.8 and 0.08 mm in radius at the bottom and top surfaces, respectively. The remnant magnetic field strength of the magnetic tip was set to produce the same magnetic field strength as the experimental setup. Similarly, the magnetic rod-magnet model consisted of an iron rod (10 mm length, 0.5 mm diameter) and a cylindrical magnet (30 mm length, 5 mm radius). This magnetic rod-magnet had a remnant magnetic field of 0.93 T in X-direction. The magnetic force on the iron sphere and rod in Z-direction was evaluated by using the force calculation function in the magnetic fields (no current module). The magnetic scalar potential at all the boundaries was assumed to be zero. The gravitational force was neglected in the simulation.

**Supplementary Table S1 List of delivered materials**

| Delivered Material | Source |
| --- | --- |
| FITC-Dextran 3-5 kDa  average mol wt 3,000-5,000 | Sigma, FD4 -100MG |
| FITC-Dextran 2000 kDa  average mol wt 2,000,000 | Sigma, FD2000s -100MK |
| TRITC-Dextran 4.4 kDa  average mol wt 4,400 | Sigma, T1037-50MG |
| Secondary antibodies | Santa Cruz, SC-362285 |
| PS bead, Lumisphere 0.2 µm | Baseline, 7-3-0020 |
| FITC-Albumin | Sigma, A9771 |
| Negative control siRNA | Life Technology |
| CXCR4 siRNA | Life Technology |
| Lamin A siRNA | Life Technology |

**Supplementary Table S2 List of primers for qPCR**

| Gene | Forward primer (5’-3’) | Reverse Primer (5’-3’) |
| --- | --- | --- |
| β-catenin | CATCTACACAGTTTGATGCTGCT | GCAGTTTTGTCAGTTCAGGGA |
| Cyclin D | CTGGAGGTCTGCGAGGAACA | CCTTCATCTTAGAGGCCACGAA |
| CXCR4 | ACTACACCGAGGAAATGGGCT | CCCACAATGCCAGTTAAGAAGA |
| GAPDH | CGCTCTCTGCTCCTCCTGTT | CCATGGTGTCTGAGCGATGT |
| p21 | GCAGACCAGCATGACAGATTT | GGATTAGGGCTTCCTCTTGGA |
| Vimentin | GACAATGCGTCTCTGGCACGTCTT | TCCTCCGCCTCCTGCAGGTTCTT |

**Supplementary Figures**

**
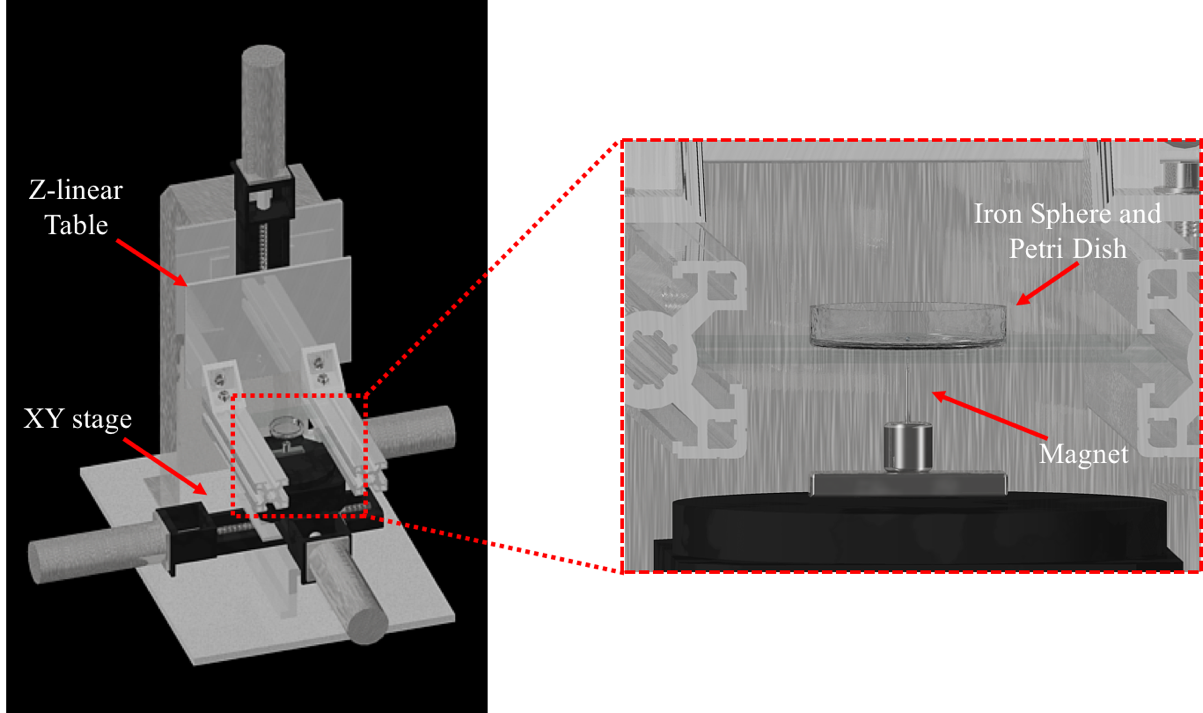
**

**Figure S1.** The experimental system for the magnetic force-driven intracellular delivery. The experimental setup included a robotic micromanipulation system with three degrees of freedom (KR30H06A, Thk Co. Ltd., 1 μm resolution), which consisted of a motorized XY stage and a Z-axis linear table. The magnet with a sharp pole tip fixed on the motorized XY stage was used to generate an effective magnetic field. A petri dish with cells was placed on the Z-axis table. Cells were loaded by actuating an iron sphere or rod via magnetic forces, which were modulated by controlling the distance between the magnet tip and the sphere/rod. The positions of the micromanipulator were robustly controlled by a motion control unit (DCT0040, Dynacity Tech. Ltd.) with a sampling frequency of 4 kHz. A CCD camera (STC-700, Sentech) and a 3× objective (Mitutoyo, Kawasaki) mounted at the two ends of an observation tube (Infinity Tube, Boulder Co.) were used for monitoring the delivery process.

**
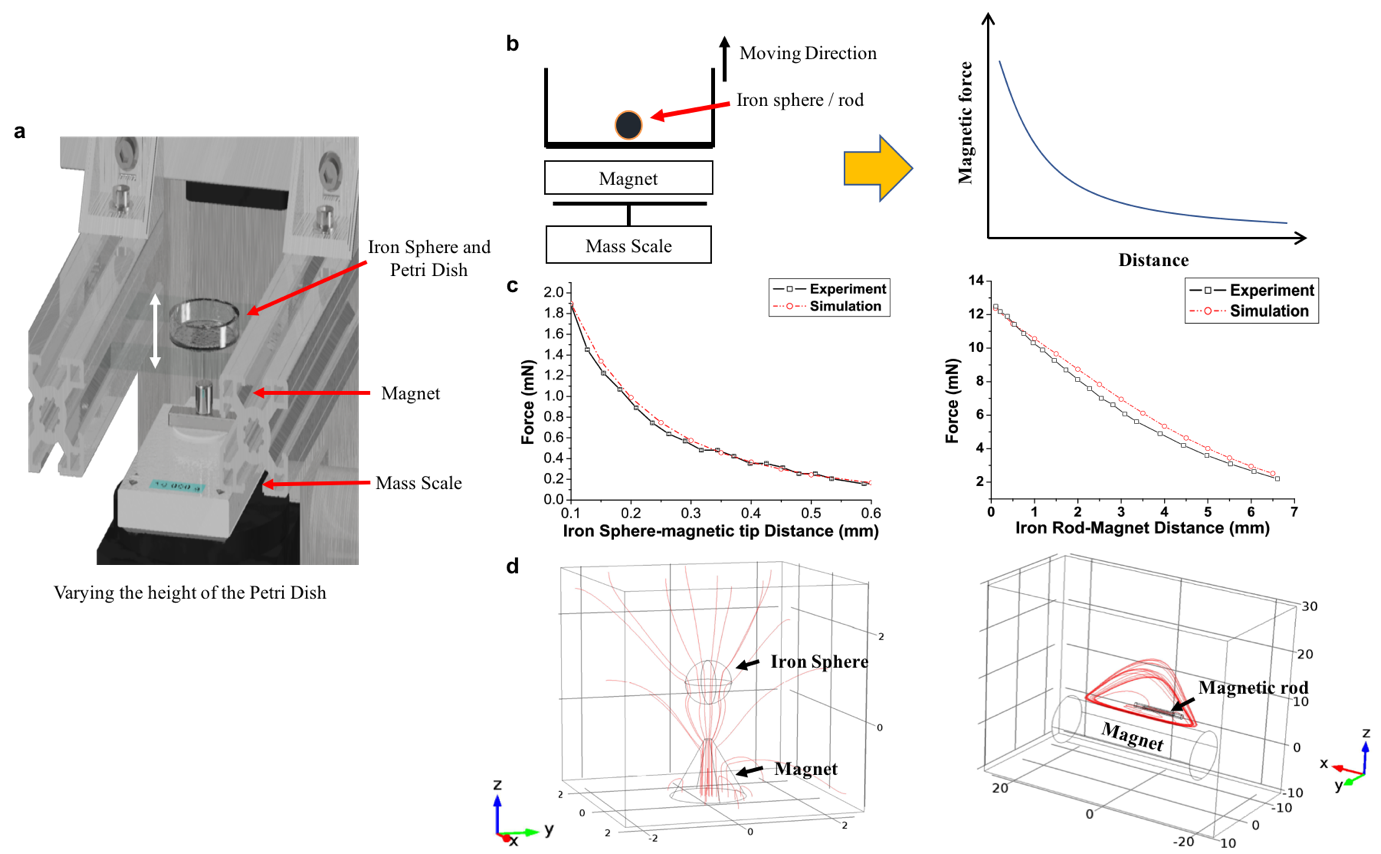
**

**Figure S2.** Calibration of magnetic forces. **(a)** The experimental setup for magnetic force calibration. The same setup described in Supplementary Figure S1 was used to calibrate magnetic forces at room temperature. **(b)** Schematic of magnetic force calibration. The magnet was placed on a mass scale fixed on the XY stage. When the sphere/rod was coupled with the magnet, the magnetic force between them partially balanced the gravitational force of the magnet, which was measured by the scale. Therefore, the magnetic force could be calculated as the decreased amount of the readout of the scale before and after the interaction. The sphere/rod-magnet distance was varied through the Z-axis table to calibrate the relationship between magnetic forces and the distances. (**c)** The relationship between the distance and magnetic forces for the sphere (left) and rod (right) derived from both experimental measurement and simulation **(d)**. The magnet-sphere/rod models were established in Comsol Multiphysics magnetic field without current module. Left: sphere-magnetic model. The sphere (1 mm in diameter) had a magnetic susceptibility of 4000. The magnetic tip (cone shape with 1.3 mm in heigh and 0.8 and 0.08 mm in radius at the bottom and top) had a remnant magnetic field of 0.335T in Z-direction. The remnant magnetic field strength of the magnetic tip was set to produce the same strength of magnetic field as in the experiments. Right: rod-magnet model. The rod was 1 mm in diameter and 10 mm in length. The magnet (cylindrical shape with 10 mm in diameter and 30 mm in length) had a remnant magnetic field of 0.93 T in X-direction. The magnetic force on the sphere and rod in Z-direction was evaluated using the force calculation function in the magnetic fields without current module). All the boundaries were assumed to have zero magnetic scalar potential. Gravity force was neglected in the simulation.


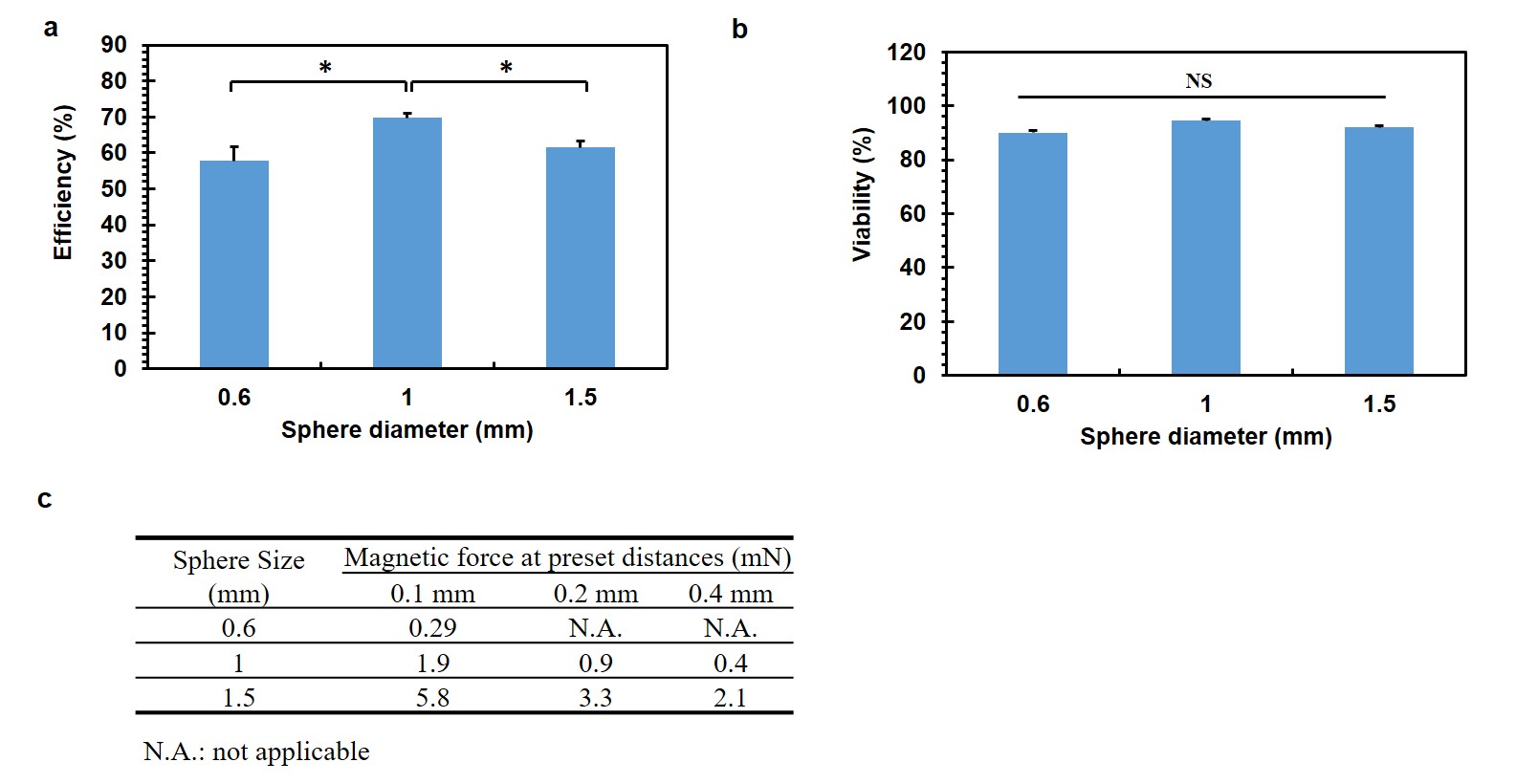


**Figure** S3. The influence of sphere size on delivery efficiency, cell viability, and magnetic force. The sphere with the diameter at 0.6, 1, and 1.5 mm was actuated to deform cells at 0.29, 1.9 and 2.1 mN in the presence of FITC-dextran. The delivery efficiency (a) and cell viability were measured based on fluorescence expression and PI staining, respectively. (c) The effects of sphere size on magnetic forces. Magnetic forces were measured following the same method in Fig. S2 for spheres with different sizes when the distance between the sphere and magnetic tip was varied.


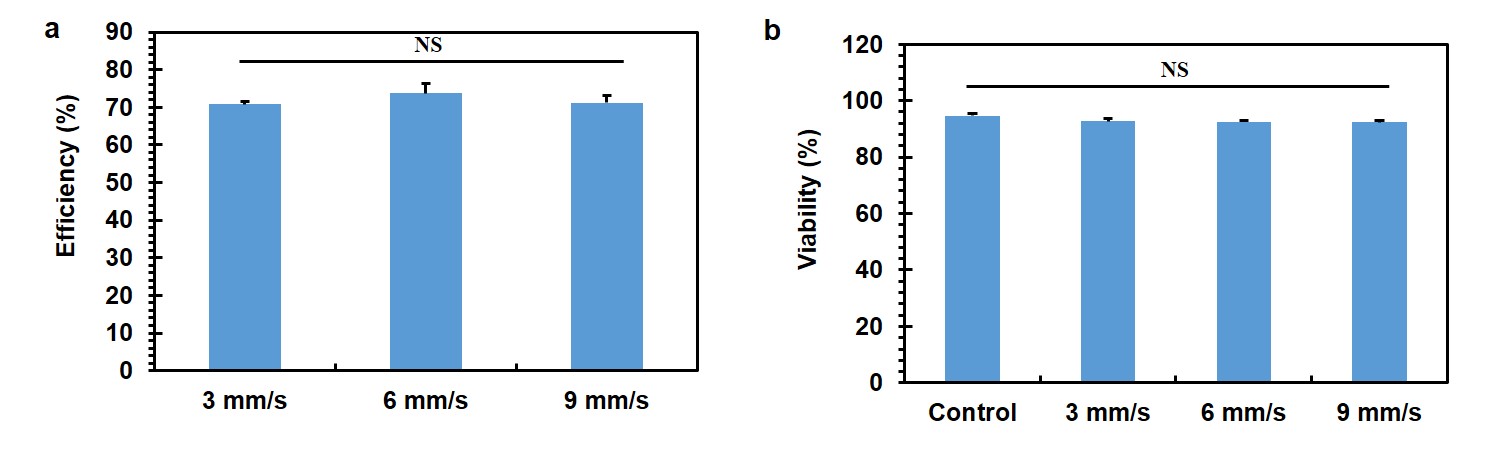


**Figure S4**. The driving speed of the sphere has no significant influence on delivery efficiency and cell viability. HepG2 cells were deformed in the presence of FITC-dextran by the sphere driven by 1.9 mN magnetic force at the speed of 3, 6, and 9 mm/s. The delivery efficiency and cell viability were quantified afterwards.


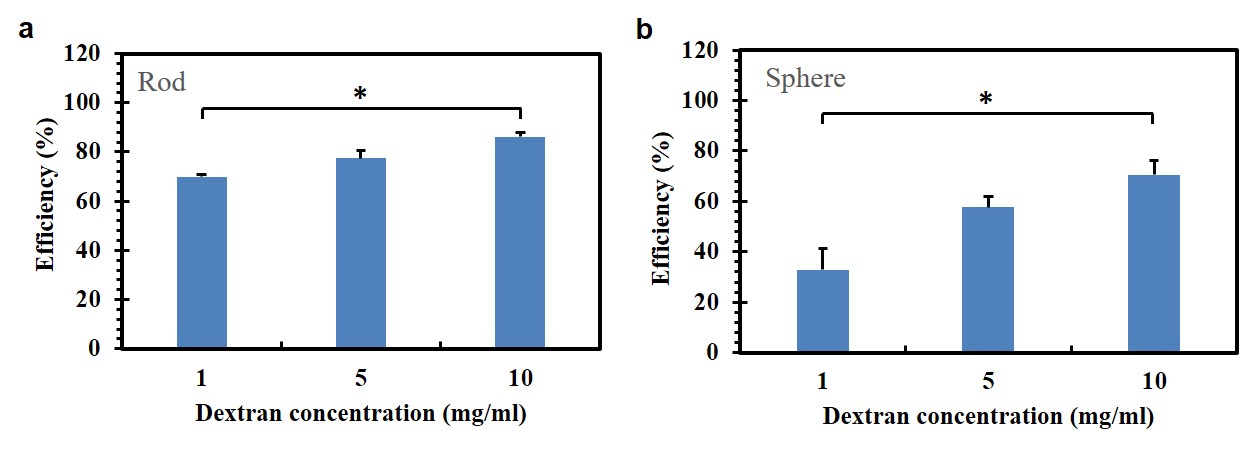


**Figure S5.** The delivery efficiency depends on molecule concentration. **(a)** Quantification of the delivery efficiency for the rod at various dextran concentrations under 12.5 mN magnetic force. **(b)** Quantification of the delivery efficiency for the sphere at various dextran concentrations under 1.9 mN magnetic force.


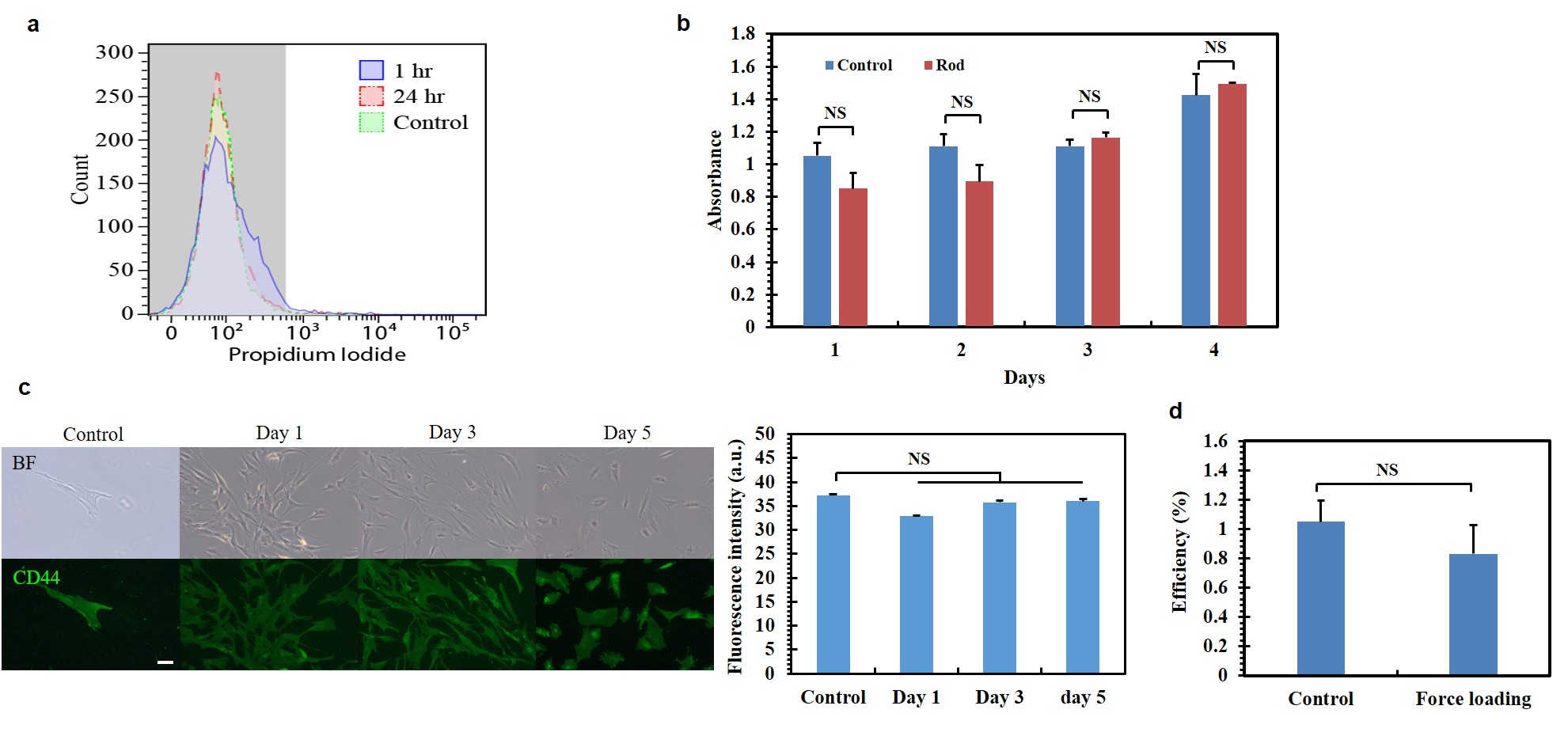


**Figure S6.** The exerted force in the intracellular delivery has minimal side effects on cell functions. **(a)** Force application has no detectable effects on cell viability. MSCs were loaded by the rod under 12.5 mN magnetic force. After 1 and 24 h, the treated cells were incubated with 500 nM propidium iodide (PI) for 5 min for flow cytometry analysis. Control: MSCs cultured in petri dishes without force application. **(b)** Force application has no significant effects on cell proliferation. The growth profiles of control and treated MSCs in (**a**) were analyzed by MTT assay at day 1, 2, 3, and 4, respectively. No significant difference was found between these two groups. **(c)** Force application has no detectable effects on CD44 expression in MSCs. Immunostaining analysis on CD44 expression (a typical MSC marker) was conducted in the treated cells in (a) at day 1, 3, and 5, respectively. The fluorescence intensity was quantified in the right panel. Scale bar: 50 µm. **(d)** The exerted force during intracellular delivery does not induce permanent defects in the membrane permeability. HepG2 cells were loaded (Force loading) by the rod under 12.5 mN force. After 3 h, these cells were incubated with 3-5 kDa FITC-dextran. The delivery efficiency was measured by flow cytometry after 24 h. Control: cells without force loading.


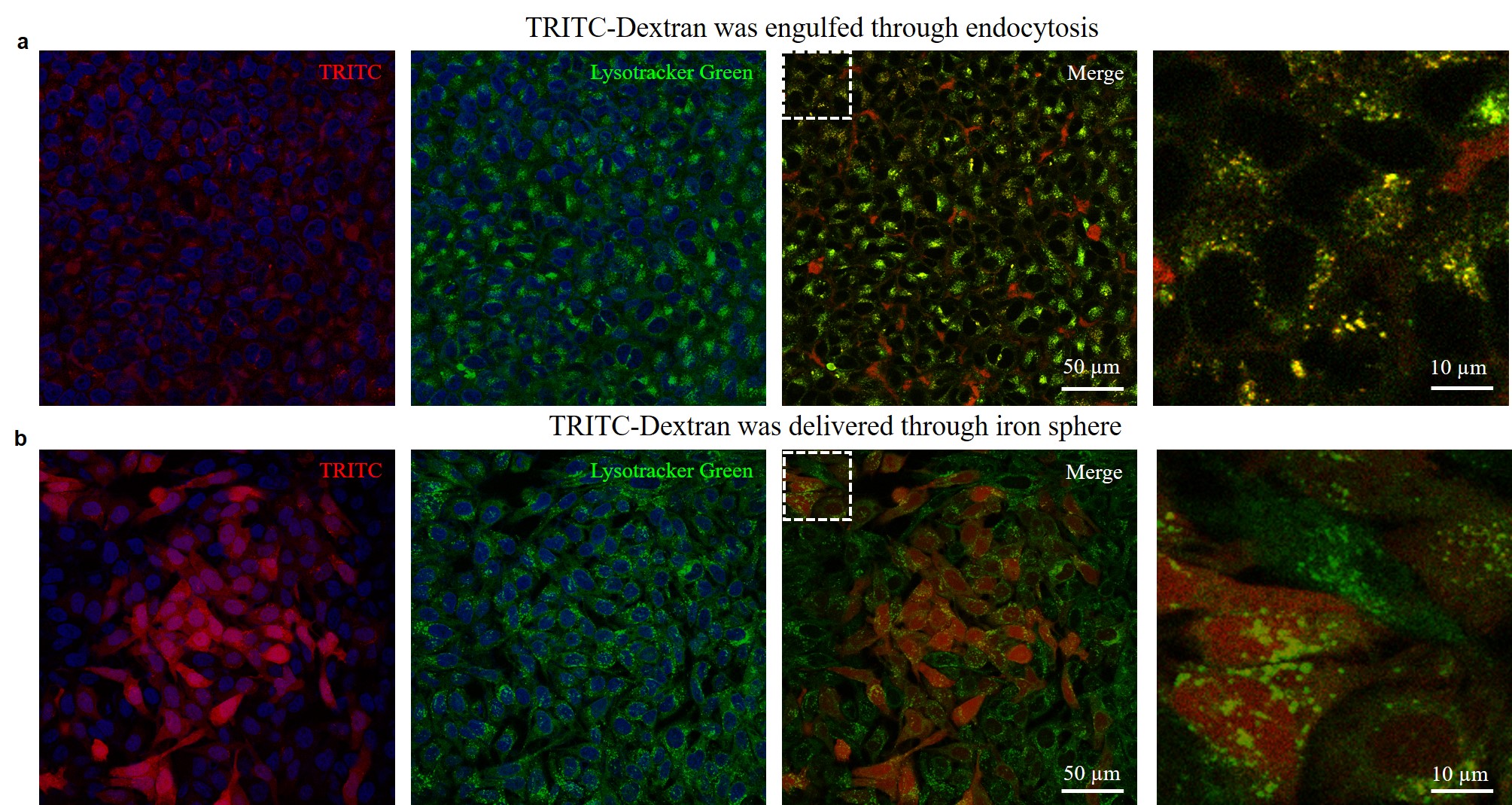


**Figure S7.** The intracellular distribution of nanoparticles delivered by magnetic force-driven approach and endocytosis. (a) Nanoparticles delivered through endocytosis are distributed in lysosomes. HepG2 cells were cultured with 10 µg/mL TRITC-Dextran (red fluorescence) for 24 hours without force loading. Lysosome was then stained with the lysotracker DND-26 (Cat. #L7526, Invitrogen; green fluorescence). (b) Nanoparticles delivered through the iron sphere are distributed in cytoplasm not lysosomes. The images in the dashed square in the third column were enlarged as shown in the last column.

**
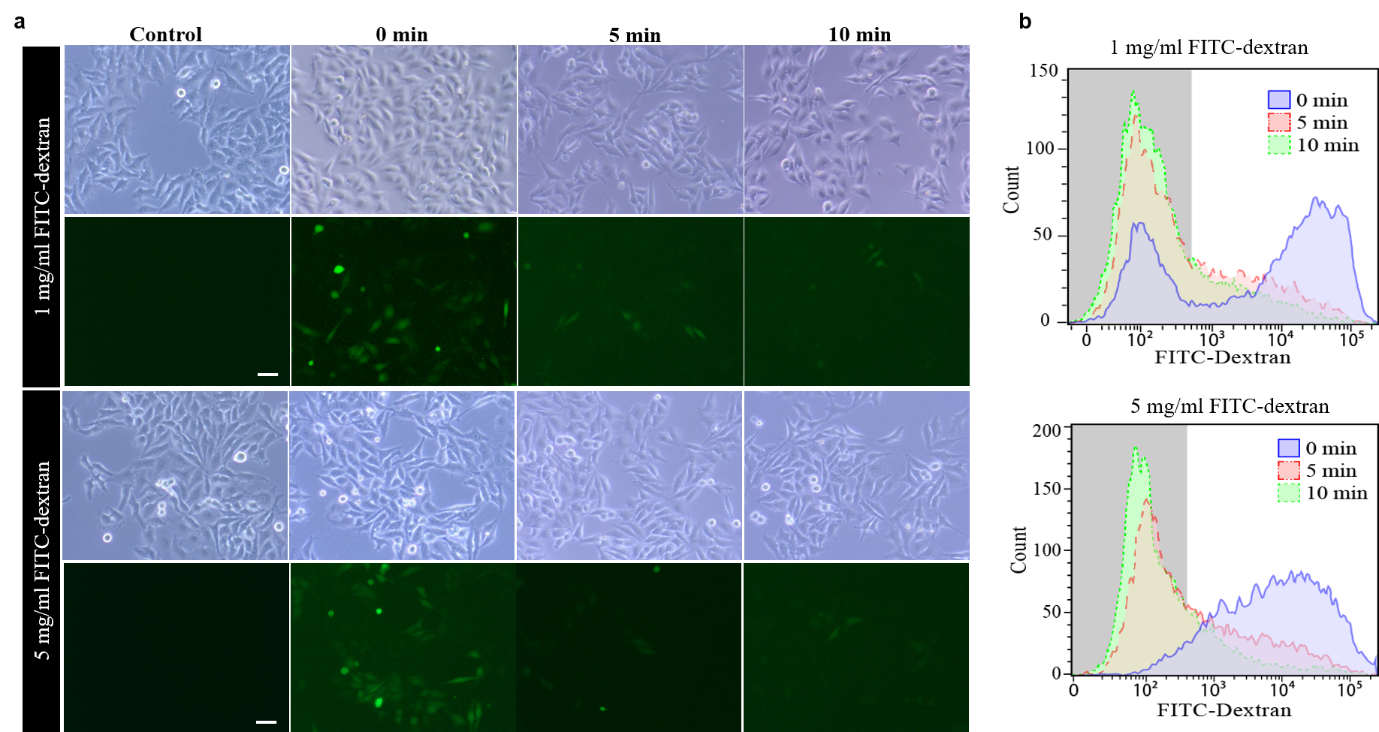
**

**Figure S8.** The delivery efficiency decreases along with the time after force application when the delivery materials are added. **(a)** The delivery efficiency dramatically decreases when 1 and 5 mg/ml FITC-dextran are added at 0, 5, and 10 min after force application, respectively. HepG2 cells were loaded by the rod under 12.5 mN magnetic force when 1 and 5 mg/ml FITC-dextran was added at 0, 5, and 10 min. The fluorescence images were taken after 24 h. Control: HepG2 cells were incubated with FITC-dextran but with no force application. Scale bar: 50 µm. **(b)** Representative flow cytometry figures show the decrease in delivery efficiency in (a).


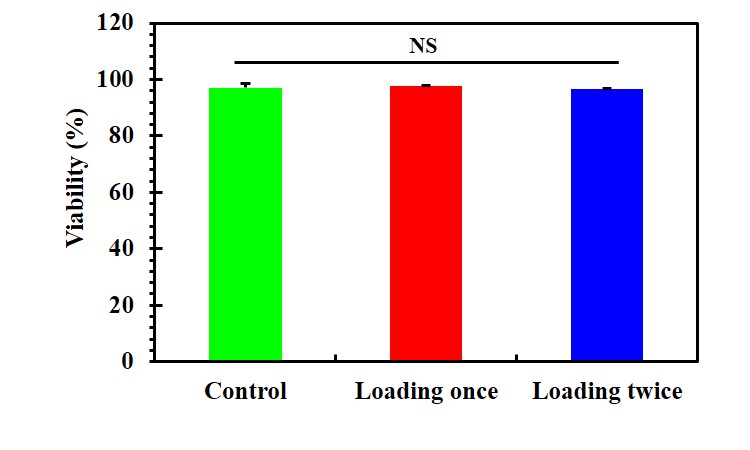


**Figure S9.** Repetitive force loading has no significant effects on cell viability. The viability of the cells in Fig. 2b was assessed by PI staining and measured by flow cytometry. NS: no significant difference.


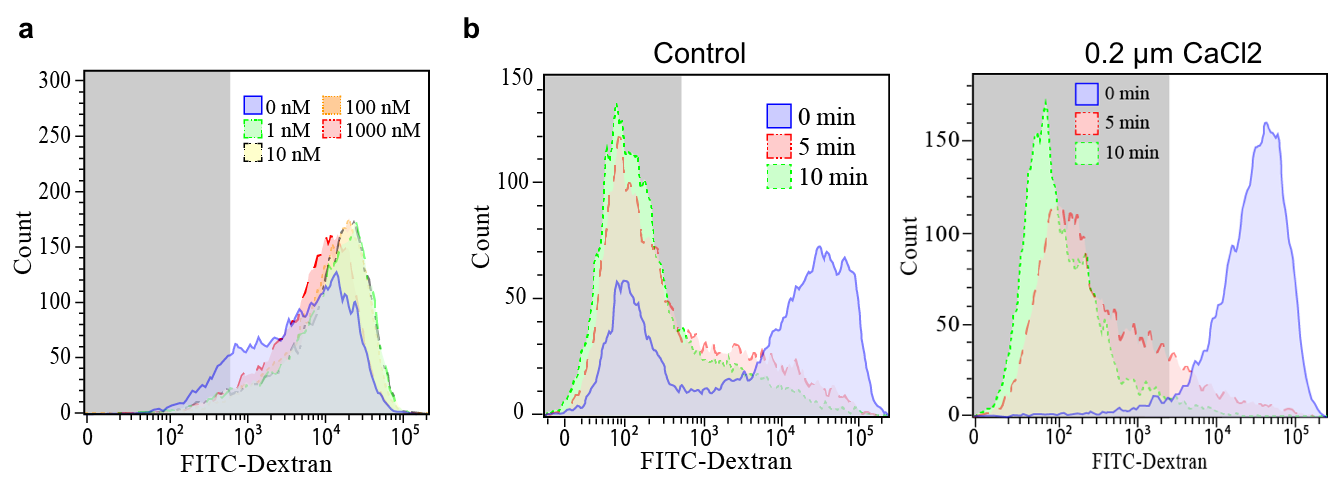


**Figure S10.** Calcium signaling facilitates membrane recovery. **(a)** Calcium signaling has no detectable effects on the delivery efficiency regardless of the concentration when FITC-dextran is added at 0 min. HepG2 cells were loaded by the rod under 12.5 mN magnetic force in the presence of 0, 1, 10, 100, and 1000 nM CaCl_2_, respectively, when the FITC-dextran was added at 0 min. The treated cells were analyzed by flow cytometry to quantify the delivery efficiency. **(b)** Calcium signaling facilitates membrane recovery and decreases the delivery efficiency. HepG2 cells were loaded by the rod under 12.5 mN magnetic force in the presence and absence (Control) of 200 nM CaCl_2_ when 3-5 kDa FITC-dextran was added at 0, 5, and 10 min, respectively. The treated cells were analysis by flow cytometry after 24 h. Note that for the convenience of reading, Supplementary Figure S8b (top) was re-used here as the left figure in (b).


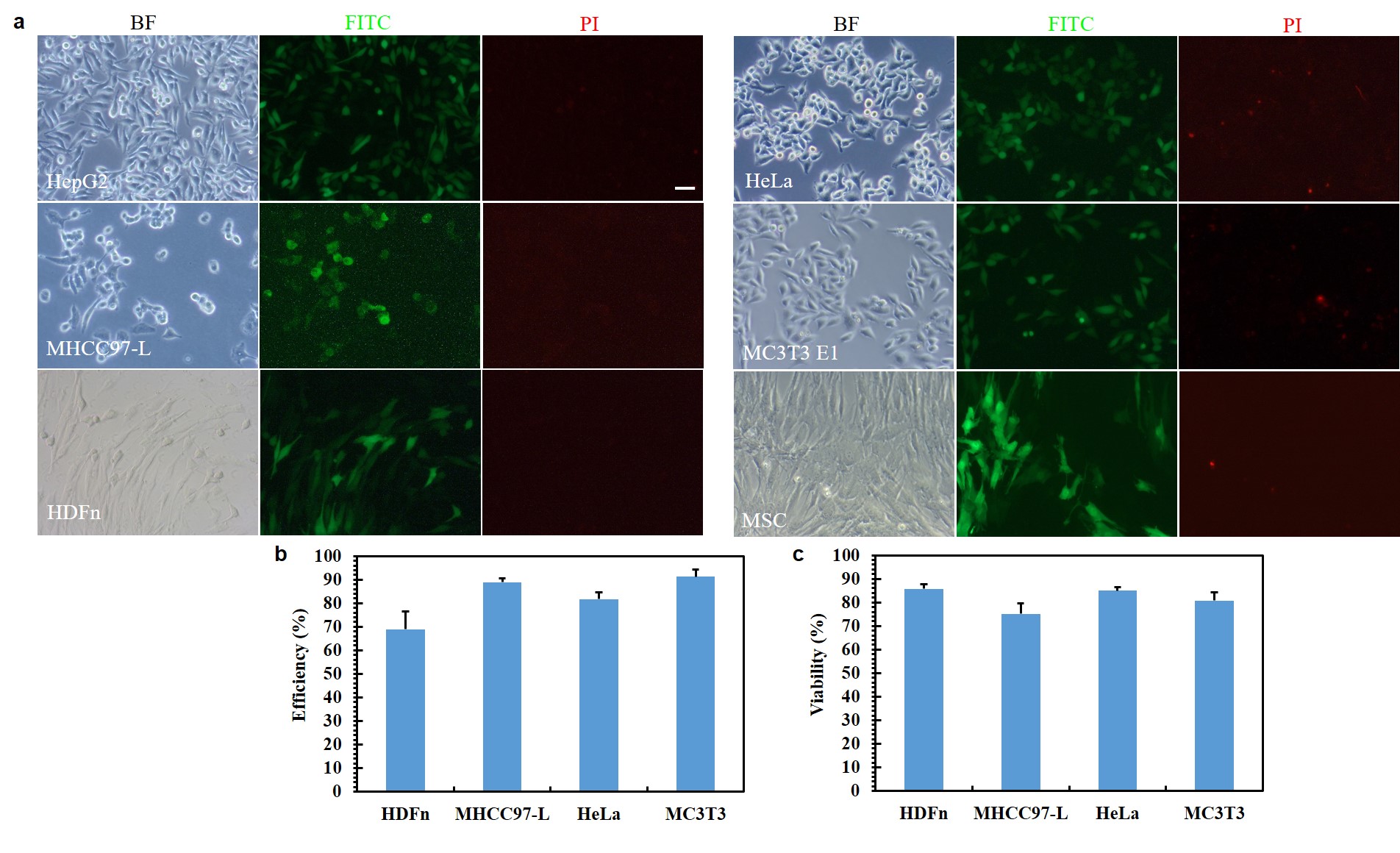


**Figure S11.** Exogenous materials are successfully delivered into various mammalian cells with high efficiency and viability. Various mammalian cells, including HepG2, MHCC97-L, human dermal fibroblasts, neonatal (HDFn), HeLa, preosteoblast MC3T3 E1, and primary human MSCs, were loaded by the rod under 12.5 mN magnetic forces in the presence of 1 mg/ml FITC-dextran. After 24 h, the treated cells were incubated with 500 nM PI for 15 min before the fluorescence images were taken (a). The delivery efficiency (b) and cell viability (c) were quantified. Scale bar: 50 µm.
